# Assessing Work-Related Burnout and Job Satisfaction among Obstetrics and Gynecology Residency Program Coordinators

**Published:** 2019-02-26

**Authors:** Samuel Ofei-Dodoo, Gretchen Irwin, Zachary Kuhlmann, Rick Kellerman, Stacey Wright-Haviland, Michaela Dreiling

**Affiliations:** 1 University of Kansas School of Medicine-Wichita, Department of Family and Community Medicine; 2 Wesley Family Medicine Residency; 3 Department of Obstetrics and Gynecology

**Keywords:** professional burnout, job satisfaction, internship and residency, administrative personnel, obstetrics and gynecology departments

## Abstract

**Introduction:**

This study explored the prevalence of and the relationship between job satisfaction and burnout among obstetrics and gynecology residency program coordinators.

**Methods:**

This cross-sectional study involved members of the American Program Managers of Obstetrics and Gynecology. The Copenhagen Burnout Inventory and Spector’s Job Satisfaction Survey were used to measure the participants’ burnout and job satisfaction rates respectively. Data were collected between August 2017 and December 2017. The authors used Fisher’s exact tests, Spearman’s *r* correlations, and multiple linear regression to analyze the data.

**Results:**

There was an 83% (171/207) response rate. Thirteen percent of the coordinators reported high, 70% moderate, and 17% low job satisfaction scores. Thirty-nine percent of the coordinators reported high, 25% moderate, and 36% slight work-related burnout rates. Correlation coefficient showed a significantly negative relationship between job satisfaction and work-rated burnout, (*r_s_*[169] = −0.402, p < 0.01). Regression analysis showed co-workers (β = −0.47) and supervision (β = −0.16) domains of the job satisfaction scale were significant predictors of work-related burnout (*R* = 0.55; *F*[5, 195] = 11.05; p < .001).

**Conclusions:**

The findings highlight the importance of job satisfaction factors, such as support from coworkers and supervisors, in dealing with work-related burnout among residency coordinators.

## INTRODUCTION

Obstetrics and gynecology (Ob-Gyn) residency program coordinators play an integral role in day-to-day operations of Ob-Gyn graduate medical education. There are different job titles of Ob-Gyn residency coordinators including program administrator, residency manager, residency program manager, and resiliency coordinator. For consistency purposes, we used “residency program coordinators” to refer to all the Ob-Gyn coordinators irrespective of their job title. Residency program coordinators have multiple roles and responsibilities that include providing administrative support to program directors, faculty, fellows, and residents; scheduling and assisting with program accreditation; and maintaining files and databases that contain faculty, fellow, and resident information. These coordinators often work in an environment that can be stressful.^1^,^2^ Increased job responsibilities could result in increased job stress if these coordinators do not receive an adequate level of institutional support.^3^,^4^

A study that included 56 residency coordinators from 21 specialties, including obstetrics and gynecology, revealed 72% were overwhelmed by job duties and responsibilities and 39% considered quitting their job.^5^ Similar sentiments were shared by family medicine residency coordinators where 81% reported they are overwhelmed by their workload and 71% indicated their work wore them out.^6^

Burnout has been associated with long-term exposure to work-related stress^7^,^8^ and low job satisfaction.^9^,^10^ Job satisfaction is multi-faceted, but can be broken into nine meaningful domains: satisfaction with pay, opportunities for promotion, fringe benefits, contingent rewards, supervision, co-workers, nature of work, communication, and work conditions. These domains are the basis for the commonly used job satisfaction survey.^11^ Work-related burnout is “the degree of physical and psychological fatigue and exhaustion that is perceived by a person as related to his/her work.”^12^

Even though burnout rate among physicians is studied widely, a review of the literature indicated little information regarding job satisfaction and work-related burnout studies involving Ob-Gyn residency program coordinators who play a pivotal role in postgraduate medical training. Therefore, the purposes of this study were to:

1. explore the prevalence of job satisfaction and work-related burnout among Ob-Gyn residency program coordinators;
2. assess if there is a relationship between job satisfaction and work-related burnout among the Ob-Gyn residency program coordinators. We hypothesized that the coordinators who are satisfied with their jobs will report low work-related burnout scores on the burnout scale; and
3. determine predictors of Ob-Gyn residency program coordinators’ work-related burnout using job satisfaction domains: pay, promotion, supervision, co-worker, and nature of work.

## METHODS

### Study Design and Participants

This cross-sectional study included data from Ob-Gyn residency program coordinators who were active members of the American Program Managers of Obstetrics and Gynecology (APMOG). Among other functions, the APMOG is a professional organization dedicated to professional growth of residency program coordinators. Study participants completed an anonymous, 43-item online survey that included questions from the Copenhagen Burnout Inventory^12^ and the Spector’s Job Satisfaction Scale,^11^ as well as questions used to construct the demographic profile of the participants. The University of Kansas School of Medicine-Wichita Institutional Review Board granted exemption for the study. A sample size of 100 was calculated as necessary for adequate power (> 0.85) to detect significant correlations of 0.5, p < 0.01 between variables.^13^

### Study Instruments

#### Job Satisfaction

The first outcome measure for the study was job satisfaction, which was defined as pleasure people derive from their work, including their ability to affect the lives of people through work positively.^14^ The job satisfaction measure was assessed using the Job Satisfaction Scale,^11^ which is a validated research tool used widely. The Job Satisfaction Scale has a set of nine domains designed to measure employee attitudes about their job and aspects of the job that include Pay, Promotion, Supervision, Fringe Benefits, Contingent Rewards (performance based rewards), Operating Procedures (required rules and procedures), Co-workers, Nature of Work, and Communication.^11^ Based on the research goal, the current study utilized statements from five of the nine domains to include:

**Pay.** This job satisfaction domain consists of four statements that measure workers perception of the pay and remuneration they receive from the work they do. An example of a statement under this domain is, “I feel I am being paid a fair amount for the work I do.”
**Promotion.** The Promotion domain of the job satisfaction has four statements that measure how workers perceive promotion opportunities they have at work. An example of a statement under this domain is, “Those who do well on the job stand a fair chance of being promoted.”
**Supervision.** This domain has four statements that measure workers perceptions of their immediate supervisor. An example of a statement under this supervision construct is, “My supervisor shows interest in the feelings of subordinates.”
**Co-workers.** This job satisfaction domain measured workers perceptions of the people with whom they work. It consists of four statements. An example is, “People I work with get along very well.”
**Nature of Work.** The four statements under this domain measured the participants’ job tasks. An example of a statement under this domain is, “I feel a sense of pride in doing my job.”

For each domain, respondents recorded how much a statement applied to them using a 6-point Likert scale ranging from disagree very much to agree very much. The scores from all the five domains were summed to form the overall job satisfaction score with higher scores indicating high job satisfaction. Consistent with convention,^15^ the job satisfaction composite score was categorized into low (< 53), moderate (53 – 88), and high (> 88).

#### Burnout

The second outcome measure for the study was burnout, which can be associated with a “very high workload or a non-supportive work environment”.^8^ The personal and work-related burnout sub-scales of the Copenhagen Burnout Inventory were used to measure the respondents’ burnout scores. Personal burnout is a state of prolonged physical or psychological fatigue and exhaustion. Work-related burnout occurs when the degree of the physical and emotional exhaustion is attributed to one’s work.^12^ By comparing the personal burnout and work-related burnout, individuals who attributed their fatigue to personal factors, such as family demands, were identified. The Copenhagen Burnout Inventory is used worldwide and has been validated in samples of administrative staff.^12^

For personal burnout, respondents recorded how often they experience physical or psychological fatigue and exhaustion using a 5-point Likert scale ranging from never/almost never to always. The overall personal burnout score is the average of the scores on the items with higher scores indicating higher degree of burnout on the personal burnout scale. Consistent with convention,^16^ the personal burnout composite score was categorized into slightly (< 50), moderate (50 – 70), and high (> 70).

For work-related burnout, respondents reported how often or the degree to which they experience work-related physical or psychological fatigue and exhaustion using a 5-point Likert scale ranging from never/almost never/to a very low degree to always/to a very high degree. One item was reverse scored. The overall work-related burnout score was computed as the average of the scores on the items with higher scores indicating higher degree of burnout on the work-related burnout scale. Consistent with convention,^16^ the work-related composite score was categorized into slightly (< 45), moderate (45 – 60), and high (> 60).

### Data Collection

There are 277 accredited obstetrics and gynecology programs nationally.^17^ Due to logistical reasons (i.e., the inability to know the total number of the Ob-Gyn residency program coordinators and their contact information), the current study included only active members of the APMOG at the time of the study. SurveyMonkey^®^ was used to host the survey and a generated link was sent via email to all of the 207 registered members of the APMOG. Data were collected between August 2017 and December 2017.

### Statistical Analyses

Standard descriptive summary statistics were used to examine the respondents’ job satisfaction rates, burnout prevalence, and to create demographic profiles of the respondents. Fisher’s Exact tests were conducted to determine the relationship among variables (job satisfaction, work-related burnout, years on the job, gender [male vs female], residency program type, and location of program). Correlations determined association between the variables, and multiple regression using the five domains of the Job Satisfaction Scale determined the best predictors of the respondents’ work-related burnout. A statistical critical value of 0.05 was specified for all tests.

## RESULTS

Of the 207 Ob-Gyn residency program coordinators surveyed, data from 171 were collected, a response rate of 83%. As shown in Table 1, 94% of the respondents were females. Thirty-nine percent of the respondents have been in their current job for less than five years; 52% were working in university-based programs; and 61% of the programs were located in urban areas. Fisher’s Exact tests showed no significant relationship among the variables.

### Job Satisfaction Results

Overall, 13% of the Ob-Gyn residency program coordinators reported high, 70% moderate, and 17% low job satisfaction scores (Table 2).

### Burnout Results

The burnout results are presented in Tables 2 and 3. Thirty-nine percent of the Ob-Gyn residency program coordinators reported high, 25% moderate, and 36% slight work-related burnout scores (Table 2). In particular, 56% of participants reported a high or very high score in the category measuring the degree to which participants were worn out at the end of the working day. Further, 39% reported high or very high scores describing their work as emotionally exhausting and 36% felt burnout because of their work (Table 3).

As shown in Table 3, 64% of the coordinators reported that they often/always feel tired; 52% often/always feel emotionally exhausted; and 52% often/always feel worn out. The overall work-related score positively correlated with the overall personal burnout score (*r_s_*[169] = 0.913, p < 0.01; Table 4).

### Job Satisfaction and Burnout Results

To test the study’s hypothesis that Ob-Gyn residency program coordinators who are satisfied with their job would report fewer symptoms of work-related burnout, a correlation coefficient was calculated. The results showed a statistically significant negative relationship between the variables (*r_s_*[169] = −0.402, p < 0.01; Table 4), suggesting that the Ob-Gyn residency program coordinators who reported satisfaction with their jobs scored low on the work-related burnout scale. As shown in Table 4, the overall work-related burnout score negatively correlated with two job satisfaction domains: co-workers (*r_s_*[169] = −0.460, p < 0.01) and supervision (*r_s_*[169] = −0.163, p < 0.05). Multiple linear regression analysis was conducted to determine the job satisfaction factors that best predicted work-related burnout. The results showed 30% of the variance was explained by the model. Co-workers (β = −0.47) and supervision (β = −0.16) domains were significant predictors of work-related burnout (*R* = 0.55; *F*[5, 195] = 11.05; p < .001; Table 5).

## DISCUSSION

The study provided information regarding job satisfaction and burnout among residency program coordinators who are an integral part of the residency education team. Our results indicated that moderate to high levels of burnout exist among Ob-Gyn residency program coordinators in both work-related and personal domains. Burnout was associated with high workload or non-supportive working environment.^6^ Our data showed 39% of the Ob-Gyn residency program coordinators reported the highest rates of work-related burnout and only 13% reported high job satisfaction. Job satisfaction and work-related burnout negatively correlated. These findings suggested that increased rates of burnout are associated with job dissatisfaction among the Ob-Gyn residency program coordinators, which is consistent with a study that has shown inverse relationship between burnout and job satisfaction.^6^

With co-workers and supervision domains of job satisfaction being predictors of work-related burnout with negative beta coefficient, an opportunity exists to modify the Ob-Gyn residency program coordinators working environment to improve factors causing the job dissatisfaction and high work-related burnout scores. An area of attention should include providing an environment that fosters positive feeling about work, good working relationships, support, and teamwork from co-workers. Consistent with findings of a previous study, co-workers and supervision domains are related most closely to job satisfaction scores and have shown to be best predictors of work-related burnout among nonclinical workers in a medical education center.^15^

Additionally, focusing on factors that contribute to emotional exhaustion may improve burnout scores for these coordinators. Emotional exhaustion has been demonstrated to be a major contributor of burnout among faculty,^18^ suggesting that faculty development programs to address this may be applicable to residency program coordinators as well. Residency program coordinators may anticipate growth in job responsibilities as graduate medical education continues to emphasize outcomes-based performance metrics and increased tracking of data for each individual resident and fellow.^19^,^20^ The additional job responsibilities could contribute to higher burnout and lower job satisfaction if programs do not analyze current stressors and workload and adjust accordingly to promote the wellbeing of residency program coordinators.^3^,^15^

The study has limitations. First, only members of the APMOG were included in the study and the results may not be reflective of all Ob-Gyn residency coordinators. However, our findings of Ob-Gyn program coordinators’ job satisfaction and burnout are similar to the findings of family medicine residency program coordinators’ job satisfaction and burnout.^6^ The survey also provided a single snapshot of Ob-Gyn residency program coordinator’s subjective responses. Although the study had a high response rate, the findings could be limited by self-report, and there is a possibility of response bias. The adaption of the modified job satisfaction scale also could limit the study findings. The original scale comprised of nine domains, but the modified scale used for this study included five domains.

## CONCLUSIONS

In conclusion, the findings of this exploratory study highlights the importance of job satisfaction factors, such as support from co-workers and supervisors among Ob-Gyn residency program coordinators. Given that job satisfaction and work-related burnout are related negatively, residency program coordinators should not be bystanders in wellness initiatives. Residency program coordinators’ wellness should be a priority and efforts to improve their job satisfaction be considered.

## Figures and Tables

**Table 1:** Demographic profile of participants (N = 171).

Demographic of Participants	Measure
**Sex, no. (%)**	
Male	9 (5.6)
Female	152 (94.4)
Missing	10
**Years on the job, no. (%)**	
< 2 years	17 (10.5)
2 – 5 years	46 (28.4)
6 – 10 years	31 (19.1)
11 – 15 years	25 (15.4)
16 – 20 years	20 (12.3)
21 – 25 years	13 (8.0)
≥ 26 years	10 (6.2)
Missing	9
**Residency program type, no. (%)**	
Community-based, medical school administered	13 (8.1)
Community-based, medical school affiliated	48 (29.8)
Community-based, non-affiliated	15 (9.3)
University	83 (51.6)
Military program	0 (0.0)
Other	2 (1.2)
Missing	10
**Community location of program, no. (%)**	
Suburban	39 (24.2)
Rural	24 (14.9)
Urban	98 (60.9)
Missing	10

**Table 2:** Level of burnout and job satisfaction among the respondents.

Levels	Personal Burnout Scale (PBS)^a^ (n = 162)	Work-related Burnout Scale (WBS)^b^ (n = 161)	Job Satisfaction Scale (JSS)^c^ (n = 171)
Slightly or low*	60 (38%)	60 (36%)	29 (17%)
Moderately or moderate*	57 (36%)	41 (25%)	120 (70%)
Highest or high*	42 (26%)	64 (39%)	22 (13%)

* Response category for job satisfaction scale.

a PBS level scoring: slightly burnout, < 50; moderately burnout, 50 – 70; highest burnout, > 70.

b WBS level scoring: slightly burnout, < 45; moderately burnout, 45 – 60; highest burnout, > 60.

c JSS level scoring: low satisfaction, < 53; moderate satisfaction, 53 – 88; highest satisfaction, >88.

**Table 3:** Participants’ burnout inventory: Scales, items and response frequencies.

	Response Category and Scoring						
	Never/almost never^a^ or to a very low degree^b^	Seldom^a^ or to a low degree^b^	Sometimes^a^ or somewhat^b^	Often^a^ or to a high degree^b^	Always^a^ or to a very high degree^b^		Score
	(Scoring 0)	(Scoring 25)	(Scoring 50)	(Scoring 75)	(Scoring 100)	Missing	Mean
Survey Items	%	%	%	%	%	*n*	(SD)
**Personal burnout** (α = 0.92) (N = 168)							
How often do you feel tired?^a^	2.3	5.8	28.1	45.0	18.7	-	68.1 (22.8)
How often are you physically exhausted?^a^	4.7	17.5	32.2	34.5	11.1	-	57.4 (25.7)
How often are you emotionally exhausted?^a^	2.3	10.5	35.1	36.3	15.8	-	63.3 (24.0)
How often do you think: “I can’t take it anymore”?^a^	15.9	17.1	33.5	21.8	11.8	1	48.7 (30.4)
How often do you feel worn out?^a^	4.1	13.6	30.2	38.5	13.6	2	60.0 (25.6)
How often do you feel weak and susceptible to illness?^a^	19.3	28.7	29.2	15.2	7.6	1	41.1 (29.5)
**Total average score**							**56.6 (22.2)**
**Work-related burnout** (α = 0.93) (N = 170)							
Do you feel worn out at the end of the working day?^a^	3.5	9.9	30.4	35.7	20.5	1	65.0 (25.9)
Are you exhausted in the morning at the thought of another day at work?^a^	16.4	17.5	26.9	27.5	11.7	1	50.1 (31.5)
Do you feel that every working hour is tiring for you?^a^	23.4	19.9	29.2	18.1	9.4	1	42.6 (31.7)
Do you have enough energy for family and friends during leisure time? (Reverse scored)^a^	9.4	31.6	35.7	13.5	9.9	1	45.7 (27.5)
Is your work emotionally exhausting?^b^	8.8	18.2	33.5	21.8	17.6	2	55.3 (29.8)
Do you feel burnt out because of your work?^b^	11.7	18.7	33.3	17.5	18.7	1	53.2 (31.4)
Does your work frustrate you?^b^	7.0	18.1	33.3	21.1	20.5	1	57.5 (29.8)
**Total average score**							**52.8 (24.8)**

Possible score range for all scales: 0 – 100

a = response categories for items denoted with ^a^.

b = response categories for items denoted with ^b^.

**Table 4:** Correlation coefficients of job satisfaction and burnout of respondents (N = 171).

Variables		Personal Burnout	Work-Related Burnout	Job Satisfaction
Personal burnout	Spearman’s Correlation	____		
	Sig. (2-tailed)			
Work-related burnout	Spearman’s Correlation	0.913**	____	
	Sig. (2-tailed)	0.000		
Job satisfaction	Spearman’s Correlation	−0.412*	−0.402**	____
	Sig. (2-tailed)	0.012	0.008	
Promotions	Spearman’s Correlation	−0.060	−0.016	0.684**
	Sig. (2-tailed)	0.440	0.838	0.000
Pay	Spearman’s Correlation	0.084	0.019	0.674**
	Sig. (2-tailed)	0.285	0.803	0.000
Supervision	Spearman’s Correlation	−0.58	−0.163*	0.738**
	Sig. (2-tailed)	0.094	0.036	0.000
Nature of work	Spearman’s Correlation	0.011	0.053	0.528**
	Sig. (2-tailed)	0.885	0.496	0.000
Co-workers Mean	Spearman’s Correlation	−0.424**	−0.496**	0.263**
	Sig. (2-tailed)	0.000	0.000	0.001
		56.6	52.8	68.8
Standard deviation		22.3	24.8	17.8
Range		0 – 100	0 – 100	20 – 120

** Correlation is significant at the 0.00125 (0.01/8) level (2-tailed).

* Correlation is significant at the 0.00625 (0.05/8) level (2-tailed).

**Table 5:** Summary statistics: Results from regression analysis.

	Work-Related Burnout				
Variables	*M*	*SD*	*b*	*b*	95% CI for *b*
(Constant)	102.33	82.42, 122.24			
Promotion	7.7	4.1	0.03	0.01	−0.99, 1.05
Pay	8.8	5.1	0.11	0.02	−0.71, 0.93
Supervision	17.2	6.5	−0.61	−0.16*	−1.19, −0.03
Nature of work	18.9	4.7	0.37	0.07	−0.41, 1.14
Co-workers	18.0	4.4	−2.63	−0.47**	−3.39, −1.88
*F*			11.05**		
*R*			0.55		
*R^2^*			0.30		

M = Mean, SD = Standard deviation

* p < 0.05,

** p < 0.001
